# Upadacitinib improved alopecia areata in a patient with atopic dermatitis: A case report

**DOI:** 10.1111/dth.15346

**Published:** 2022-02-10

**Authors:** Mariateresa Cantelli, Fabrizio Martora, Cataldo Patruno, Paola Nappa, Gabriella Fabbrocini, Maddalena Napolitano

**Affiliations:** ^1^ Section of Dermatology ‐ Department of Clinical Medicine and Surgery University of Naples Federico II Naples Italy; ^2^ Department of Health Sciences University Magna Graecia of Catanzaro Catanzaro Italy; ^3^ Department of Medicine and Health Sciences Vincenzo Tiberio University of Molise Campobasso Italy

Dear Editor,

Atopic dermatitis (AD) and alopecia areata (AA) are two common diseases that can coexist in the same patient, sharing some pathogenetic aspects such as the overexpression of T‐helper (Th) 2 cytokines interleukin‐4 (IL‐4) and IL‐13, the altered expression or loss‐of‐function mutations of atopy related genes in patients affected by AA (such as filaggrin), and finally the elevated serum levels of immunoglobulin E in both diseases.^1^, ^2^, ^3^ For these reasons, the use of dupilumab, a fully monoclonal antibody directed against the IL‐4 receptor α subunit and approved for the treatment of adults and adolescents with moderate‐to‐severe AD, has recently been proposed for the treatment of AA in patients with or without AD.^1^, ^4^ However, its effectiveness in AA treatment is controversial.^1^ Conversely, Janus kinase inhibitors (JAKs) 1 seem to constitute a new therapeutic option in the management of both AD and AA.^5^

We present the case of a 24‐year‐old patient with an history of AD since childhood and 10 years of AA, who comes to our outpatient after the failure of topical and systemic therapies, including corticosteroids and cyclosporine. On physical examination the patient had severe AD, involving his hand, trunk, lower, and upper limbs [Eczema Area Severity Index (EASI): 45.1; Pruritus‐Numeric Rating Scale (P‐NRS): 8/10)]. Patches of AA were located on his scalp, eyebrows, and eyelashes with a Severity of Alopecia Tool (SALT) score of 89.2. Since the failure of first‐line therapies and the concomitance of the two diseases we decided to start treatment with dupilumab.^1^, ^4^ At the 4‐month follow‐up visit, despite the marked improvement in the body skin lesions, the patients showed erythemato‐squamous lesions in the head and neck area, associated to itching and burning and resistant to treatment with potent topical corticosteroids and antifungal medication. A paradoxical “red face” due to dupilumab was diagnosed and dupilumab was stopped. AA remained unchanged during the 4 months of treatment (SALT: 91.5). Then, we decided to start therapy with upadacitinib 30 mg/die (Rinvoq®), an oral selective JAK‐1inhibitor recently approved by the European Medicine Agency for the treatment of moderate‐to‐severe AD of adults and adolescents 12 years and older candidates to systemic therapy.^6^

Before starting therapy, laboratory tests were performed, including complete blood count, liver and kidney function test, hepatitis markers, and screening tests for tuberculosis. As there is several case reports and clinical trials reporting promising outcomes of JAK‐inhibitors also for AA,^5^, ^7^ at week 0 a clinical and trichoscopical examination of the scalp was performed showing the presence of numerous yellow dots, some black dots and broken air (Figure 1A–C) After 3 months treatment, we noticed both clinical improvement of both AD and AA (Figure 1D–F). Trichoscopy shows regrowing hair all over the scalp without any sign of disease activity. No adverse events have reported. JAKs are multidomain non‐receptor tyrosine kinases that have pivotal roles in cellular signal transduction.^8^ The JAK and signal transducer and activator of transcription (STAT) pathway has been shown to play an essential role in the dysregulation of immune responses in AD, including the amplification of Th2 cell response, the eosinophils activation, the maturation of B cells, the suppression of regulatory T cells, and the upregulation of pro‐inflammatory cytokines and pro‐angiogenic factors.^8^ Concerning AA, laboratory studies have focussed on to JAK/STAT signaling to amplify the inflammatory response around hair follicles.^8^ So, the JAK/STAT signaling pathway represents a potential target in patients affected by AD, AA, or both.^8^ However, also in relation to the recent reporting of the Food and Drug Administration,^9^ more studies are needed to confirm both the efficacy and safety of JAK‐inhibitors.

**FIGURE 1:**
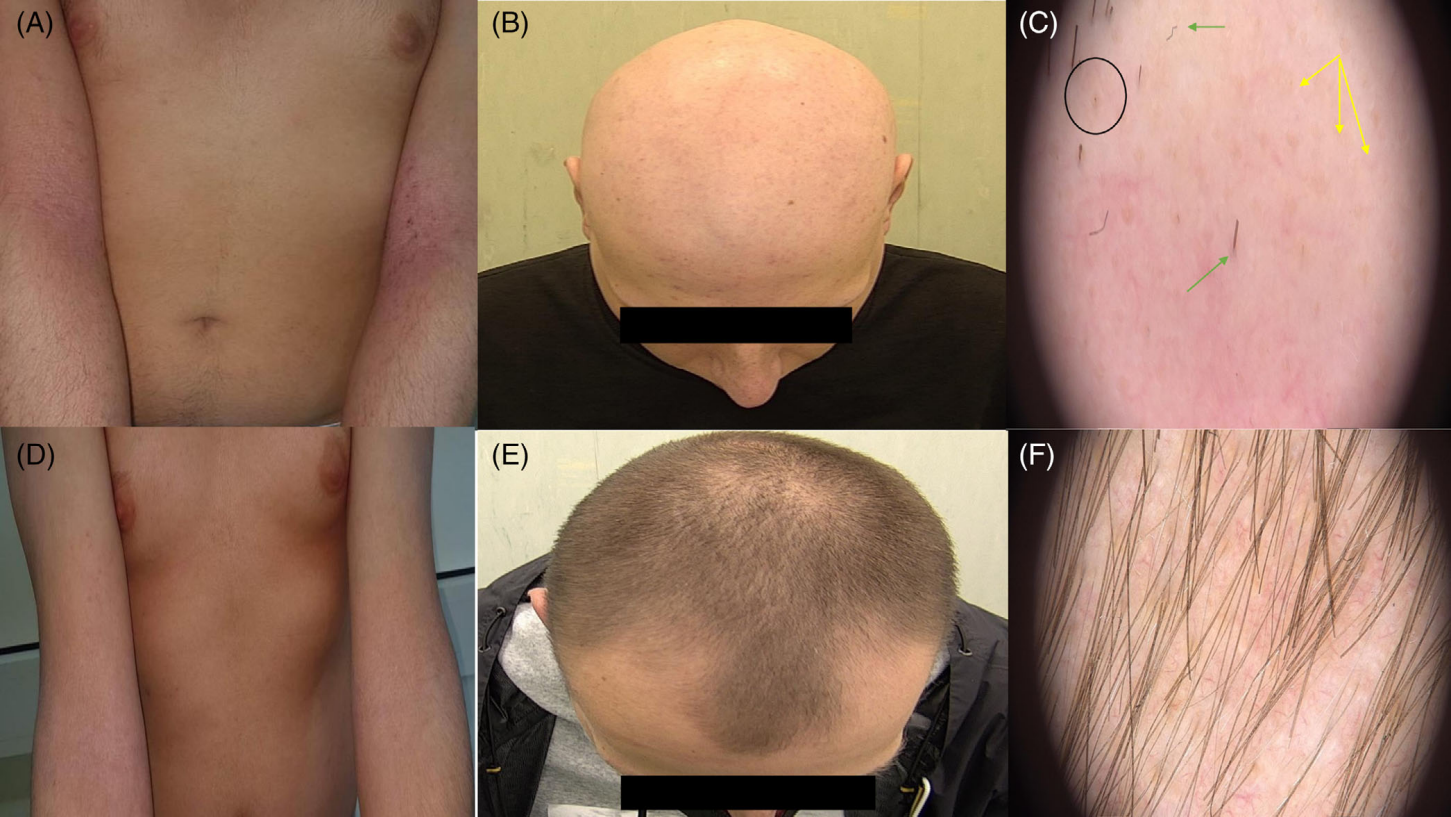
(A) Eczematous and crusted lesions involving upper limbs at week 0. (B, C) Clinical and trichoscopical examination of alopecia areata of our patient at week 0. (D) Resolution of skin lesion of upper limbs after 3 months of treatment with upadacitinib. (E, F) Clinical and trichoscopical examination of alopecia areata after 3 months of treatment with upadacitinib

## CONFLICT OF INTEREST

For this work AbbVie provided the study drug Upadacitinib (Rinvoq) through a Compassionate Use Program activated according to the DM 7/9/2017.

Patruno C. acted as investigator, speaker, consultant, and advisory board member for AbbVie, Eli Lilly, Novartis, Pfizer and Sanofi; Gabriella Fabbrocini is a member of the journal's Editorial Board, and has been principal investigator in clinical trials sponsored by and/or and has received personal fees from AbbVie, Abiogen, Almirall, Celgene, Eli‐Lilly, Leo Pharma, Novartis, Sanofi, and UCB; Napolitano M acted as speaker, consultant and advisory board member for Sanofi, Abbvie, Leo Pharma and Bionike; Cantelli M, Martora F, Nappa P have nothing to disclose.

## AUTHOR CONTRIBUTIONS

Mariateresa Cantelli: conceptualization, validation, visualization, writing—original draft preparation, writing—review & editing. Fabrizio Martora: conceptualization, validation, visualization, writing—original draft preparation, writing—review & editing. Cataldo Patruno: conceptualization, validation, visualization, writing—review & editing, supervision. Paola Nappa: validation, visualization. Gabriella Fabbrocini: conceptualization, validation, visualization, writing—review & editing, supervision. Maddalena Napolitano: conceptualization, validation, visualization, writing—original draft preparation, writing—review & editing. All authors read and approved the final version of the manuscript.

## ETHICS STATEMENT

The patient in this manuscript has given written informed consent to publication of their case details.

## Data Availability

The data that support the findings of this study are available from the corresponding author upon reasonable request.
